# Gene Expression Profiling as a Tool for Positional Cloning of Genes-Shortcut or the Longest Way Round

**DOI:** 10.2174/138920208786241180

**Published:** 2008-11

**Authors:** Lena W Rosenlöf

**Affiliations:** Section for Immunogenetics, University of Rostock, Germany

## Abstract

The identification of quantitative trait loci, QTL, in arthritis animal models is a straight forward process. However, to identify the underlying genes is a great challenge. One strategy frequently used, is to combine QTL analysis with genomic/proteomic screens. This has resulted in a number of publications where carefully performed genomic analyses present likely candidate genes for their respective QTL´s. However, seldom the findings are reconnected to the QTL controlled phenotypes. In this review, we use our own data as an illustrative example that “very likely candidate genes” identified by genomic/proteomics is not necessarily the same as true QTL underlying genes.

## 1. INTRODUCTION

Despite last decade’s therapeutic progression, Rheumatoid arthritis (RA) still lacks cure and satisfactory treatment in many cases. To make a change, it is urgent to elucidate the underlying disease mechanisms. As RA has a genetic component of ~60% [1], one way to go is to identify predisposing genes. The strongest genetic contribution to RA comes from the HLA region, which is expected to count for about 35% of the genetic predisposition [2]. However, to learn more about underlying mechanisms also additional contributing genes need to be identified. Unfortunately, this has turned out to be far from easy as the genetics behind RA is complex, i.e. heterogeneous, polygenetic with low penetrance of each contributing gene, epistatic and with interaction between genes and environment. As the human population is outbred and exposed to highly variable environment, it is really difficult to reproducibly, identify predisposing genes in human material. Also, phenotypes necessary for the understanding of disease mechanisms (e.g. disease on-set and progression from acute to chronic inflammation) cannot be studied in humans as most patients once they get their diagnosis already are in a chronic state of disease. In an attempt to overcome these problems, we and others use animal models. Animals can be genetically and environmentally controlled, genetically manipulated and the disease can be followed in detail before on-set until the progression into chronic disease.

## 2. FIRST STEP OF GENE IDENTIFICATION –QTL IDENTIFICATION

Firstly, to identify RA relevant genes in rodents, a disease model with high similarity to the human disease has to be chosen. Several such models have been developed; e.g. induction by injection of cartilage proteins in adjuvant oils [3] or by injection of adjuvant oils only (in rats) [4]. A drawback of some models is that they only result in acute self-healing arthritis, but some models e.g. autologous collagen II induced arthritis, aCIA, and pristane-induced arthritis, PIA, in rats [5] are also suitable for studies on chronic relapsing arthritis. In resemblance to RA these models are highly dependent on the genetic background, with MHC as the most important individual region, but with obvious contribution also of other genes [5, 6]. The assumption is that these other genes also need to be identified in order to understand RA. To do so, a first step is to identify chromosomal loci controlling quantitative arthritic traits (quantitative trait loci, QTL). This is done with genome-wide linkage analyses in crosses between susceptible and resistant strains. Nowadays, QTL identification is pretty strait forward and ~70 joint/bone inflammation QTL´s have been identified for the rat [7] and almost 400 arthritis QTL´s for the mouse [8]. However, the subsequent step, to identify the underlying gene(s), has turned out to be challenging. So far, only a small number of genes have been identified from a QTL and then it has been genes from QTLs that have extraordinary large effect sizes [9].

## 3. STRATEGIES FOR GENE CLONING FROM A QTL

Before consider strategies for cloning a gene from a QTL, one has to be aware of what one is looking for. As has been nicely pointed out by Flint *et al*. [9] most single nucleotide polymorphisms, SNPs, underlying QTL´s for complex diseases are not likely to be found within coding-sequences. In the worse case the position of the quantitative trait nucleotide, QTN does not coincide with the position of the gene it is affecting (trans-regulated genes). However, let us assume that the causative variant of a gene indeed is encoded from the QTL. Then, to work out strategies for its identification it is important to have in mind which criteria such a gene should fulfill. Suggested criteria are; the gene should be polymorphic in a way that leads to altered protein product or differential expression, its expression should be detected in organs or cell types of relevance and the gene shall have a function either related to the trait directly or in a biologic pathway of relevance for the trait [10, 11]. However, a typical QTL identified in a F2-cross encodes too many genes to allow criteria fulfillment analysis of all the genes. Therefore, the QTL need to be narrowed to reduce the possible candidates to a number that can be handled for investigation. To narrow a QTL, one can choose between safe, “hard-core evidence” strategies and “likelihood” based strategies. The former are often expensive, laborious and time-consuming sometimes even beyond the border of be performable at all. The second strategy is not so safe but can more easily be afforded, economically as well as time- and effort-wise.

### 3.1. Safe, “Hard-Core Evidence” Strategies

The classical way of gene identification is to construct a congenic strain where the QTL region of the F2-cross parental strains “A” has been substituted with the corresponding region from parental strain “B”. This is done by systematic breeding for 6-10 generations depending on method [12], to receive a” B”- derived QTL genomic fragment on a pure “A” background genome. If the phenotype can be reproduced in the congenic animal, one can conclude that the affecting polymorphism indeed is encoded from this region. To narrow the causative fragment using the congenic animals, the animals are further backcrossed and genotyped. Off-springs where a genomic recombination has occurred, resulting in a smaller “B” –derived congenic fragment, are tested for the disease phenotype. Congenics positive for the phenotype are selected for further backcross. This method is safe when systematically performed and has successfully been used in cloning of the arthritis susceptibility gene *Ncf1* [13]. However, to be feasible, as discussed below, it assumes that the QTL underlying gene has a strong, dominant, sex-independent effect on the phenotype. In addition, this strategy is time consuming as it is dependent of recombinant events to occur, which cannot be forced. The only possibility to speed up the procedure is to provide resources for a large breeding and simultaneous phenotype-testing of many subcongenics. Another strategy to collect recombination is to produce advanced intercross lines (AIL) [14]. In these lines recombinations are collected by continue the intercross several generations beyond F2. AIL´s has been successfully used to narrow down several QTLs including QTL´s for autoimmune diseases in mice and rat [15-18]. An extension of this strategy is the uses of heterogeneous stocks (HS) that are AIL with several parental strains [19]. A disadvantage of these strategies is that they require time and space for (production)/hosting and phenotyping the lines as well as facilities for high-trough-put genotyping.

### 3.2. More Easily Affordable “Likely Hood” Strategies

As most of the “safe” hard core evidence strategies have large requirements of infra structure and economical resources many scientists turn to more easily performable “likely hood”-strategies. Even it may be argued that these strategies are more ethically correct as they more quickly can provide data for validation in humans and require fewer experiment animals. 

The cheapest, but often foreseen, method is to use bioinformatics tools to narrow rodent QTL´s [20]. Already a lots of data (especially for the mouse) are available in data bases all over the world and if correctly analyzed they can save a lot of time, money and laboratory efforts. An interesting, combined strategy for identifying QTL underlying genes with bioinformatics tools have been suggested in [20]. This includes comparative genomics, combined cross analysis, interval specific haplotype analysis, sequence comparison and expression data base analysis.

An intermediate strategy effort and economically wise, is to make congenes and use genomic and proteomic screens to narrow the QTL. This strategy is aimed to, in a nearly unbiased way, find genes within the QTL that fulfills the differential qualitative protein product and differential expression criteria for QTL underlying genes. This strategy have gained popularity after some nice publications showing that the strategy indeed works and gives candidate genes that can be referred back to the QTL phenotype [21-24]. In the light of this we also tried this strategy with our QTL *Pia6* that specifically control the chronic phase of PIA.

## 4. BACKGROUND OF THE DA.PIA6 CONGENE

The PIA6 QTL was identified in an F2-cross between the PIA susceptible DA and PIA resistant E3 rat [6]. The region was specifically associated with arthritis chronicity increasing in importance over time (no of affected paws day83, LOD score 4.2 and day 120, LOD score 4.9). The inheritance was DA recessive with E3 as the protective parental. The variance explained by the locus was 16-19% and the peak marker was D14Csna (21.9 MBp) and flanking markers were approximately at 15 MBp and 45 MBp (positions according to reference sequence, NCBI). We produced a congenic rat, DA.Pia6 with speed congenic technique. A ~50Mp fragment between D14Wox8 (19.1 MBp) and D14rat64 (68.6 MBp) from the E3 rat was introgressed on a DA background. This congenic rat was shown to specifically protect from chronic arthritis [25]. Using this rat, the underlying gene should be possible to clone and generate essential knowledge of chronic inflammation specific processes.

Previously, in our lab, it was shown that a gene, *Ncf1*, underlying a QTL *Pia4* indeed could be positionally cloned using a strategy of recombinant selection where the disease phenotype was used as selection criteria [13]. However, several important things differ between the Pia4 and Pia6. The Pia4 phenotype, which quickly is evaluated, could be seen in relatively few heterozygous individuals of both sexes. This meant that if a new recombinant rat was a male it could produce enough of individuals for testing the disease phenotype in one generation, =19weeks^1^ and then the phenotype would take additional 3 weeks to test. In the Pia6 rat, heterozygous rats of both sexes most likely are protected, but a strong, significant phenotype using a reasonable number of rats can only be seen in homozygous, male rats. Practically, this means that a new recombinant Pia6 rat would need to be bred to produce off-springs: =19weeks, then the off-springs need to be intercrossed to produce homozygous rats: = additional 19 weeks. As only males can be used and in a higher number than in the Pia4, eventually one more amplifying breeding step has to take place: = additional 19 weeks. Then it would be possible to test the new recombination for the disease phenotype, which for the Pia6 rat takes 12-15 weeks to evaluate. Accordingly, the same procedure which took 22 weeks for the Pia4 rat would take 12-18 months for the Pia6 rat! Obviously, recombinant selection, using the disease as phenotype, was not a plausible strategy to identify the gene underlying *Pia6.* One alternative strategy could be to investigate immunological subphenotypes of importance for the disease phenotype and use them for testing the recombinant fragments (subphenotype assisted recombinant selection). Another strategy could be to narrow down a fragment surrounding gene/genes with functions likely to be important for regulation of (chronic) inflammation (candidate gene assisted recombinant selection). However, immunological subphenotyping of healthy and arthritic rats in various disease phases only gave one obscure subphenotype; differential plasma protein concentration of alpha-1-acid glycoprotein and alpha-1-microglobulin [25] and the genetic region did not encode any immunological genes of obvious interest. Therefore, we turned to the use of genomic and proteomic screens to detect candidate genes in the Pia6 region.

## 5. EXPRESSION PROFILING STRATEGY TO NARROW DOWN PIA6

Initially we performed an Affymetrix screen of healthy and immunized rats from the background strains DA and E3 and searched for candidates within the Pia6 regions [26]. However, the only differentially expressed gene mapping to rat chromosome 14 was *Bst1* and this was positioned *too* far away from the Pia6 peak to be a reasonable candidate. Therefore, we decided to make a new affymetrix screen with tissues from the DA.Pia6 rat and DA rat in the late chronic phase of PIA, day 127 after immunization (and also healthy rats of both strains as control). We choose to analyze spleen, lymph nodes and paws and then also liver because of our plasma protein phenotype. We analyzed the tissue on U34A chips and ended up with ~50 differentially expressed genes (Change p-value 0 for increased and 1 for decreased according to Affymetrix software) per tissue and time point for the liver and lymph nodes, ~half the number in spleen and double the number in paws. The resulting list of genes we compared to genes positioned in the interval 19.1-68.9 MBp on rat chromosome 14 according to NCBI. 19 of the differentially expressed genes (all tissues) were encoded from the fragment (Table **1**) and thereby fulfilled one criterion for being candidates for the Pia6 underlying genes, differential expression. Most of the genes were position between 19-22 MBp, which correlated well with the original linkage analysis peak D14csna (21.9MBp) a group of three genes were positioned around 33-34 Mbp and a third group was positioned around 45MBp. The linkage analysis peaks gene, casein alpha, was differentially expressed. This could be an interesting observation as casein expression in lymph nodes is correlated to recovery from Experimental autoimmune encephalomyelitis, EAE [27]. This is especially interesting since the Pia6 also controls the number of relapses in EAE [28]. Another interesting candidate in this region was the splice factor YT521, which is involved in alternative splicing of soluble interleukin 4-receptor [29]. The most promising candidate in the 33-34 Mbp region is Kdr as it is essential for angiogenesis which plays an important role in arthritis [30]. The differentially expressed genes around 45MBp is regarded as less likely candidates as they are positioned on the QTL flanking border.

## 6. PROTEOMIC STRATEGY TO NARROW DOWN PIA6

**Fig. (1). 2D gel analysis of plasma from DA and DA.PIA6 rats.**
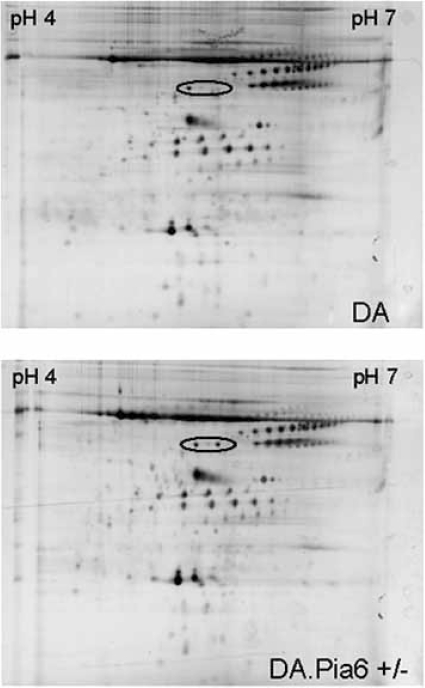
Plasma from healthy and chronically ill DA and DA.Pia6 rats were separated by charge on pH4-7 Immobiline dry strips and separated for size on 12% SDS-PAGE. All samples were repeated three times. According to analysis there was one major difference between the DA and DA.Pia6, the single (DA) and duplicated spot (DA.Pia6+/-) (marked with a ring). According to mass-spectrometry these dots were identified as two isoforms of the vitamin D binding protein encoded from the Gc locus.

QTL underlying genes do not necessarily have to show differential expression, but can show qualitative differences in the protein product. Such differences might be detectable on a 2D gel. Therefore, in parallel with the Affymetrix experiment, we did 2D gel screens of the DA and DA.pia6 rats. As we had a robust, reproducible phenotype in the differential concentration of plasma lipocalin proteins, we decided to analyze plasma from healthy and chronically ill DA and DA.Pia6 rats. After repeating each gel run at least three times per animal and time point, we could observe on major spot difference between DA and heterozygous DA.Pia6 rats both in healthy and chronically ill animals (Fig. **1**). The DA rat had one spot in this position whereas the DA.Pia6 rat had two. After extraction, digestion and mass-spectrometry analysis of the resulting peptides we could identify the spots as isoforms of vitamin D binding protein most likely representing different glycoforms. The vitamin D binding protein is encoded from the Gc locus positioned at 20.2 MBp, very close to the top marker in the linkage analysis (Csna, 21.9). Interestingly, vitamin D binding protein plays an important role in regulation of neutrophils and activation of macrophages [31-33]. Accordingly, both regarding its mapping position and its known function it was a very likely candidate for the gene underlying the Pia6 QTL.

## 7. VALIDATION OF THE STRATEGIES-RESULTS FROM SUBCONGENES

**Fig. (2).**
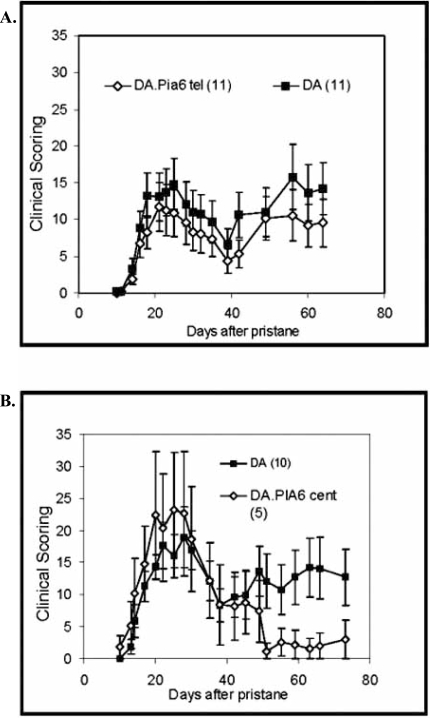
PIA experiment of DA and telomeric and centromeric DA.Pia6 subcongenic rats. Pristane was injected in DA, telomeric DA.Pia6 and centromeric DA.Pia6 rats and the animals were followed for clinical signs of arthritis for 75 days. The telomeric subcongenic of DA.Pia6 (**A**, ◊) does not have any effect on the arthritis, while the centromeric DA.Pia6 subcongenic (**B**, ◊) is protected in the chronicle, relapsing phase of arthritis.

Subsequently, after our two large screens we had a handful of really good candidates for the Pia6 QTL underlying genes. However, to validate our genomic/proteomic strategy we run a PIA experiment with two, still large, subcongenics basically dividing our original congene in two overlapping parts; a telomeric part (borders 19.1 MBp-33.5 MBp (d14rat14)) and a centromeric part (borders 68.9MBp -32.6MBp (d14rat8)). According to the position of the original linkage analysis peak, most genomic candidate genes and our proteomic candidate gene, we expected that the telomeric fragment would exert the effect. However, to our great surprise we could not detect any arthritis protective effect in the telomeric subcongene (Fig. **2A**). On the opposite, a protective effect was found in the centromeric fragment (Fig. **2B**). Surprised by reality, we were skeptical and repeated the arthritis experiment several times, but the outcome was the same: The effect was indeed in the centromeric part. This excluded the vitamin D binding protein and all genomic candidates except, *Rn.21892* and *Kdr, Ppat* and *Paics* (eventually as they are positioned on the subcongenic fragment border) and *Uchl1*, *Ugdh* and *Klf3* (eventually, positioned too far away from the original linkage analysis peak). Though, *Kdr* is a promising candidate gene to investigate further, the result highlights the risks of the genomic/proteomic strategy in narrowing a QTL. A likely gene is not always the same as a true gene and our findings clearly illustrates the necessity of reconnecting found candidates to the disease model. 

## 8. CONCLUSIONS AND SUGGESTIONS

The joint conclusion from publications in the field and our own data is that a genomic/proteomic strategy to narrow QTLs can work, but is combined with large risks to get lost. In the initial reports of genomics as a tool to narrow QTLs and in some later publications, the finding of candidate genes is reconnected to the animal models for confirmation. However, often this is not the case, and in the light of our data, it is very risky. If we had not reconnected our findings to our animal model, we would have been convinced that we had found the candidates for our QTL. If we then would, in line with many publications, have started to investigate our candidates *in vitro* we would certainly have detected functions of theoretical importance for arthritis. However, theoretical importance is not the same as true importance and we would have lost the benefit of letting the disease show us relevant genes and biological pathways rather than vice versa.

Therefore, in our opinion genomic/proteomic strategies as well as bioinformatics tools should be regarded as ways to come up with qualitative hypothesis rather than final proofs. Unfortunately, in the end, more laborious strategies like AIL or HS might be the shortest way around. If one is short in infra structure for this one may try “light version” of these strategies, nicely demonstrated to work for CIA by Yu [34] and Johannesson [35] or search for helpful collaborators.

## Figures and Tables

**Table 1:** Differentially Expressed Genes in DA and DA.Pia6 Rats Positioned within the DA.Pia6 Congenic Fragment

Position (MBp)*	Gene	Liver Day 0	Liver Day 127	LN Day 0	LN Day 127	Spleen Day 0	Spleen Day 127	Paws Day 127
19.1	Alb			↑	↑			
19.3	Rn.8442				↑			
20.5	Slc4a4		↓		↑			
20.9	Rn.165282						↓	
21.1	Ambn						↑	
21.4	Vcsa2					↓		
21.9	Csn1s1			↓	↑			
22.0	Sult1b1	↓	↓					
22.4	Ugt2b4		↓					
22.5	Rn.11131				↑			
22.6	Udpgtr2	↓						↑
22.8	Yt521			↑				
33.5	Ppat		↓					
33.5	Paics			↓			↓	
34.3	Rn.21892						↑	
34.5**	Kdr							↑
44.1	Uchl1						↑	↓
45.6	Ugdh		↑					
46.2	Klf3				↑		↑	

* Position according to the reference sequence map at http://www.ncbi.nlm.nih.gov/mapview/

** Not positioned in reference sequence, but at 31.5 in the Celera map. 34.5 is estimated according to the position of neighbor genes positioned in both Celera and reference map.

## Footnotes

1 From the birth of a rat it takes approximately eight weeks until it can be put on breeding, three more weeks until the pups are born and additionally eight weeks until the pup can be used for further breeding or PIA experiment.

## ABBREVIATIONS

aCIA: = Autologus Collagen II Induced Arthritis
AIL: = Advanced Intercross Line
CIA: = Collagen II Induced Arthritis
EAE: = Experimental Autoimmune Encephalo-myelitis
HS: = Heterogeneous Stock
LOD: = Logaritm of Odds
MBp: = Mega Base pair
MHC: = Major Histocompatibility Complex
NCBI: = National Center for Biotechnology Information
PIA: = Pristane Induced Arthritis
PIA4: = Pristane Induced Arthritis quantitative trait locus 4
PIA6: = Pristane Induced Arthritis quantitative trait locus 6
QTL: = Quantitative Trait Locus
QTN: = Quantitative Trait Nucleotide
RA: = Rheumatoid Arthritis
SNP: = Single Nucleotide Polymorphism
